# *In*-*vitro* immunomodulatory and anti-cancerous activities of biotransformed products of Dianabol through *Azadirachta indica* and its molecular docking studies

**DOI:** 10.1186/1752-153X-7-163

**Published:** 2013-10-07

**Authors:** Saifullah, Saifullah Khan, Azizuddin, Sobia Ahsan Halim, Muhammad Kashif, Almas Jabeen, Muhmammad Asif, Muhammad Ahmed Mesaik, Zaheer Ul-Haq, Ahsana Dar, Muhammad Iqbal Choudhary

**Affiliations:** 1 Department of Biotechnology, Faculty of Science, University of Karachi, Karachi 75270, Pakistan; 2 Dr. Panjwani Center for Molecular Medicine and Drug Research, International Center for Chemical and Biological Sciences, University of Karachi, Karachi 75270, Pakistan; 3 Department of Chemistry, Federal Urdu University for Arts, Science & Technology, Gulshan-e-Iqbal Campus, Karachi 75300, Pakistan

**Keywords:** Biotransformation, Anti-inflammatory activity, Anti-cancer activity, Molecular docking studies

## Abstract

**Background:**

Plant Biotransformation is one of the tools for structural modifications of the organic substrate of low, moderate or high biological value utilizing plant cultured cells, these modifications of organic structures may lead to biologically augmented products and which may be ultimately substantial in cure or improvement of various morbidities and diseases.

**Results:**

*Azadirachta indica* A. Juss. suspension culture was employed for the biotransformation of dianabol (1) for the first time, and two metabolites, 17*β*-hydroxy-17*α*-methyl-5*α*-androst-1-en-3-one (2), and 17*β*-hydroxy-17*α*-methyl-5*α*-androstan-3-one (3) were obtained.

**Conclusions:**

Most important aspect of this work was the evaluation of metabolite 2, which strongly and differentially suppressed [not affecting whole blood and human polymorphonuclear cells (PMN)] the phytohemagglutinin (PHA)-activated T-cell proliferation (IC_50_: <10.33 μM), and also found to inhibit IL-2 production (IC_50_: 16.89 ± 1.32) unlike metabolite 3 and compound 1. Compound 2 also exhibited anticancer activity against lung cancer cell line; NCI-H460, it moderately inhibited the growth of cancer cells (22.5 ± 4.15 μM). Furthermore, a good correlation between the predicted binding energies of the compounds acquired by the FlexX program and the experimental affinities were speculated upon interacting with IL-2 protein during molecular docking studies.

## Background

Plant cell suspension cultures can serve as tools for the *in vivo* production of secondary metabolites [1,2] as well as for the biotransformation of xenobiotics [3-5]. These cultures are considered to be useful biocatalysts for reactions, such as hydroxylation at allylic positions, oxidation–reduction between alcohols and ketones, and the reduction of carbon–carbon double bonds [6,7]. Plant cell cultures mediated biotransformations are now increasingly employed by synthetic chemists for the structural modifications of various organic compounds. This exhibits a vast biochemical potential for the production of specific secondary metabolites. Biotransformations, using plant cells and isolated enzymes, have an immense potential in the production of pharmaceuticals.

Plant enzyme biocatalysts may be applied to the production of totally new drugs and also may be used to modify existing drugs by improving their bioactivity spectrum. The introduction of a functional group into terpenoids and steroids is an important reaction in synthetic chemistry. Many studies have been reported on the specific oxidation, reduction of olefins, and alicyclic hydrocarbons with chemical reagents [8-10]. The ability of cultured plant cells to transform some organic compounds is useful for mass production of substances. Plant cell cultures and microbacteria are considered to be useful biocatalysts for reactions such as the hydroxylation at allylic positions, the oxidation–reduction of alcohols and ketones, and reduction of carbon–carbon double bonds [11].

Human health is directly influenced by immune system, and its performance, which are fundamentally designed for the protection against the attack of foreign invaders. However the onset of almost all infectious and degenerative diseases is largely due to inadequate or hyperactive immune response. Therefore, the modulation of the immune system is highly demanded for control of many immunological disorders. Therefore, dianabol (1) was selected for this objective, potent steroid, is a derivative of testosterone, exhibiting strong anabolic and moderate androgenic properties. This compound was first made available in 1960, and it quickly became the most favoured and widely used anabolic steroid by athletes.

In continuation of our studies [12,13], on biotransformation of molecules/compounds in pursuing of more potent and promising candidate including new drugs for different diseases, dianabol (1) was incubated with *Azadirachta indica* A Juss. cell suspension cultures, which is employed for the first time for structural modifications. *A*. *indica* has not used before for the structural modification of organic compounds except our group [13-15]. *A*. *indica* belongs to the family Meliaceae and is known to human being from 2000 years. It is generally recognized as Indian neem (margosa tree) or Indian lilac (neem in Pakistan) and inhabited in the Asia particularly in Pakistan and India. The neem is called ‘Arishtha’ in sanskrit meaning ‘reliever of sickness’. Neem importance in treating various diseases has also been distinguished by US National Academy of Sciences as they published a report entitled ‘Neem – a tree for solving global problems’ in 1992, this strongly suggested that neem is a medicinal plant possessing thousands of organic compounds, which in turn is important for playing pivotal role in combating several diseases [16]. The medicinal role of this plant evoked its biotransformational studies in hope to have various derivatives of dianabol possessing useful therapeutic activities as a result of diversified enzymes that *A*. *indica* would have, which carry out these structural modification.

Plant biotransformation of various steroids has already been described by many groups in which different reactions were observed for instances a report explains the changes in ethynodiol diacetate by the suspension culture of *Ocimum basilicum* and the same plant, in this article biotransformation with *Ocimum basilicum* performed hydrolysis of the ester group, oxidation of alcohol into ketone, and rearrangement of the hydroxyl group and with *A indica* hydrolysis of the ester group, oxidation/reduction reactions were observed [14]. Similarly, biotransformation of 21-O-acetyl-deoxycorticosterone by cell suspension cultures of *Digitalis lanata* illustrates how plant cell suspension can carry out unique reactions; 2β-Hydroxylation and C-21-glucosylation of the steroidal nucleus was being reported first time by plant-culture cells besides 5α and 5β reduction [17]. This the advantage of the cell suspension culture of plants over the synthetic methods to execute different reactions easily and cost effectively, ultimately inquiring biologically application. Both of these plant biotransformation steroid models did not mention any biological activities unlike this study. Biotransformation of dianabol afforded two metabolites 2 and 3 resulting from the reduction of olefinic double bonds, being reported for the first time by this method. The structures of transformed were deduced by various spectroscopic methods, are reporting findings on the immunomodulatory role of metabolite 2 and 3 with substrate, compound 2 is found capable of reducing the PHA dependant Th1 response induced in human peripheral mononuclear cells. The investigation of molecular characteristics of compounds 1–3 possibly associated with the inhibition of IL-2 protein was also carried out. A modeling study using FlexX program was performed to dock the compounds 1–3 with the reference inhibitor 4 into the active site of the IL-2 protein.

International patents of the metabolites 2 have recently been approved and published by United States of Patent and Trademark Office [18,19], claiming the anti-inflammatory and anti-cancer activities.

## Results

After 20 days of incubation, the cells and the media were separated by filtration. The filtrate was extracted with CH_2_Cl_2_ (3 × 1.5 L) and the cells were extracted in an ultrasonic bath with CH_2_Cl_2_ (3 × 500 mL) at r. t. The combined extract were dried over anhydrous Na_2_SO_4_, and concentrated under reduced pressures, which afforded a brown residue (1.32 g). The transformed metabolites were isolated from this crude by using repeated column chromatography (silica gel) with petroleum ether/EtOAc gradient, afforded metabolites 2 (26 mg, petroleum ether: EtOAc, 9.5:0.5, 5.2% yield) and 3 (18 mg, petroleum ether: EtOAc, 9.8:0.2, 3.6% yield). We did not find any other metabolite as column was run till the 100% of the ethyl acetate.

### 17β-Hydroxy-17α-methyl-5α-androst-1-en-3-one (2)

Colourless solid, m.p. 140–142°C, [α] D^20^ -40º (*c* 0.04, CHCl_3_). UV (MeOH) λ_max_ (log ϵ): 230 nm (3.62). IR (CHCl_3_) ν_max_: 3445 (OH), 1674 (C = O), 1652, 1496 (C = C), 1380 cm^-1^ (C-O). EI-MS *m*/*z* (rel. int. %): 302 (10), 284 (3), 245 (15), 232 (3), 200 (5), 160 (18). HREI-MS *m*/*z*: 302.0122 (*M*^+^, C_20_H_30_O_2_; calcd 302.0120). ^1^H (CDCl_3_, 300 MHz) and ^13^C NMR (CDCl_3_, 100 MHz).

### 17β-hydroxy-17α-methyl-5α-androstan-3-one (3)

Colourless solid, m.p. 152–154°C, [α]D^20^ -130º (*c* 0.02, CHCl_3_). UV (MeOH) λ_max_ (log *ϵ*): 203 nm (0.43). IR (CHCl_3_) *ν*_max_: 3360 (OH), 1713 (C = O), 1372 cm^-1^ (C-O). EI-MS *m*/*z* (rel. int. %): 304 (20), 289 (28), 271 (15), 247 (45), 231 (39), 215 (14), 189 (12), 175 (16), 163 (46). HREI-MS *m*/*z*: 304.2113 (*M*^+^, C_20_H_32_O_2_; calcd 304.2115). ^1^H (CDCl_3_, 300 MHz), and ^13^C NMR (CDCl_3_, 100 MHz).

### Anti-inflammatory activity

In this study, effects of metabolites 2 and 3 on the innate immune response, in particular the reactive oxygen species (ROS) production was examined using whole blood phagocytes, and isolated neutrophils, which did not result in any significant effect (Table 1). In addition to that, effects of these compounds on T-cells proliferation were also evaluated; investigating their ability to modulate PHA activated T-cell proliferation response and production of IL-2 cytokine.

**Table 1:** **IC**_**50 **_**effect of tested compounds on oxidative burst of whole blood, isolated PMNs, T-cell proliferation and IL-2 cytokine**

**Comp**. **Code**	**Oxidative burst IC**_**50 **_**(μM) on**		**T**- **Cell proliferation IC**_**50**_ (**μM**)	**IC**_**50 **_**IL**-**2** (**μM**)
	**Whole blood**	**PMNs**		
1	>333.33	>166.67	>166.67	>166.67
2	>331.13	>165.56	<10.33	16.89 ± 1.32
3	>328.95	>164.47	42.11 ± 11.51	49.34 ± 1.32
Control A^a^	11.2 ± 1.9	2.8 ± 0.8	-	-
Control B^b^	-	-	0.2	<0.5

^a^Ibuprofen; ^b^Prednisolone.

**Figure 1:**
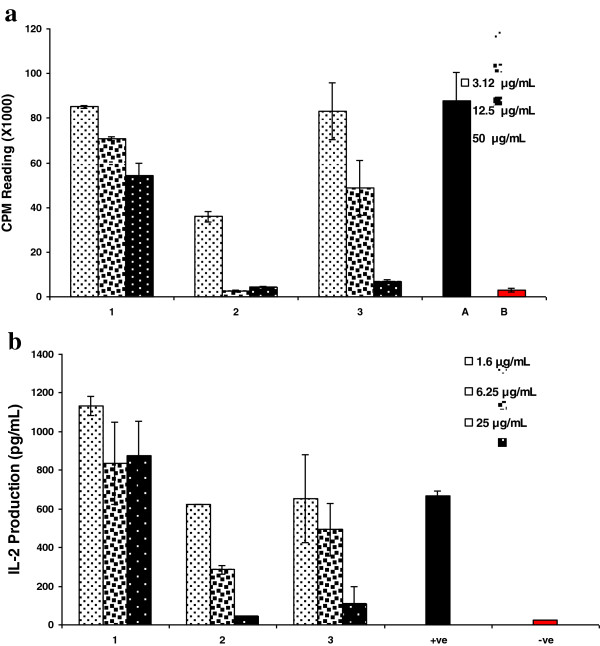
**Effect of compounds on phytohemagglutinin (PHA), T-cell proliferation and IL-2 production.** The bar graph represents effects of various concentrations of the test compounds 1–3 after 72 h incubation with peripheral blood mononuclear cells at 37°C. Effect of compounds on T-cell proliferation response is compared with non-proliferated (+ve) and proliferated (−ve) cells **(a)**. The bar graph represent effects of various concentrations of the test compounds (1–3) on production of IL-2 compared with (+ve) and without (−ve) PHA induced IL-2 production. Each bar represents the mean value of triplicate reading ± SD **(b)**.

Compound 2 was found to have significant inhibitory activity on T-cell proliferation with IC_50_ value less than 10.33 μM compared to the initial substrate dianabol (1), which did not show considerable effect neither on T-cells proliferation nor IL-2 production (Figure 1). A moderate inhibitory activity with IC_50_ value of 42.11 μM was obtained with compound 3. The activity of these two compounds was further confirmed by their effects on IL-2 cytokine production, the main contributor in T-cell activation. The extracellular production of IL-2 from peripheral blood mononuclear cells was significantly inhibited (IC_50_ = 16.9 ± 1.32 μM) by compound 2. On the other hand compound 3 had a moderate inhibitory effect (IC_50_ = 49.3 ± 1.32 μM) on IL-2 production.

### Anti-cancer activity

The biotransformed products and its substrate were also screened for the anticancer activity against NCI-H460 cell line. During the screening, compound 2 (GI_50_: 22.5 ± 4.15 μM and TGI: 60 ± 9.2 μM) and 1 (GI_50_: 28.3 ± 2.72 μM) exhibited growth inhibitory effects. The GI_50_ and TGI represents the concentration of compounds exhibiting 50% and 100% (total) growth inhibition respectively against the NCI-H460 cell line. The compound 3 was non-active against NCI-H460 cell line as it showed no growth inhibition (Table 2).

**Table 2:** Growth inhibition induced by transformed compounds against human lung cancer cell line (NCI-H460)

**Compound(s)**	**GI**_**50 **_**(μM)**	**TGI (μM)**
1	28.3 ± 2.72	ND^a^
2	22.5 ± 4.15	60 ± 9.2
3	ND	ND
Doxorubicin (control)	0.08 ± 0.02	---

^*a*^*ND*: Values could not be determined indicates <50% inhibition of cell growth at maximum dose (100 μM) tested.

### Docking studies

**Figure 2:**
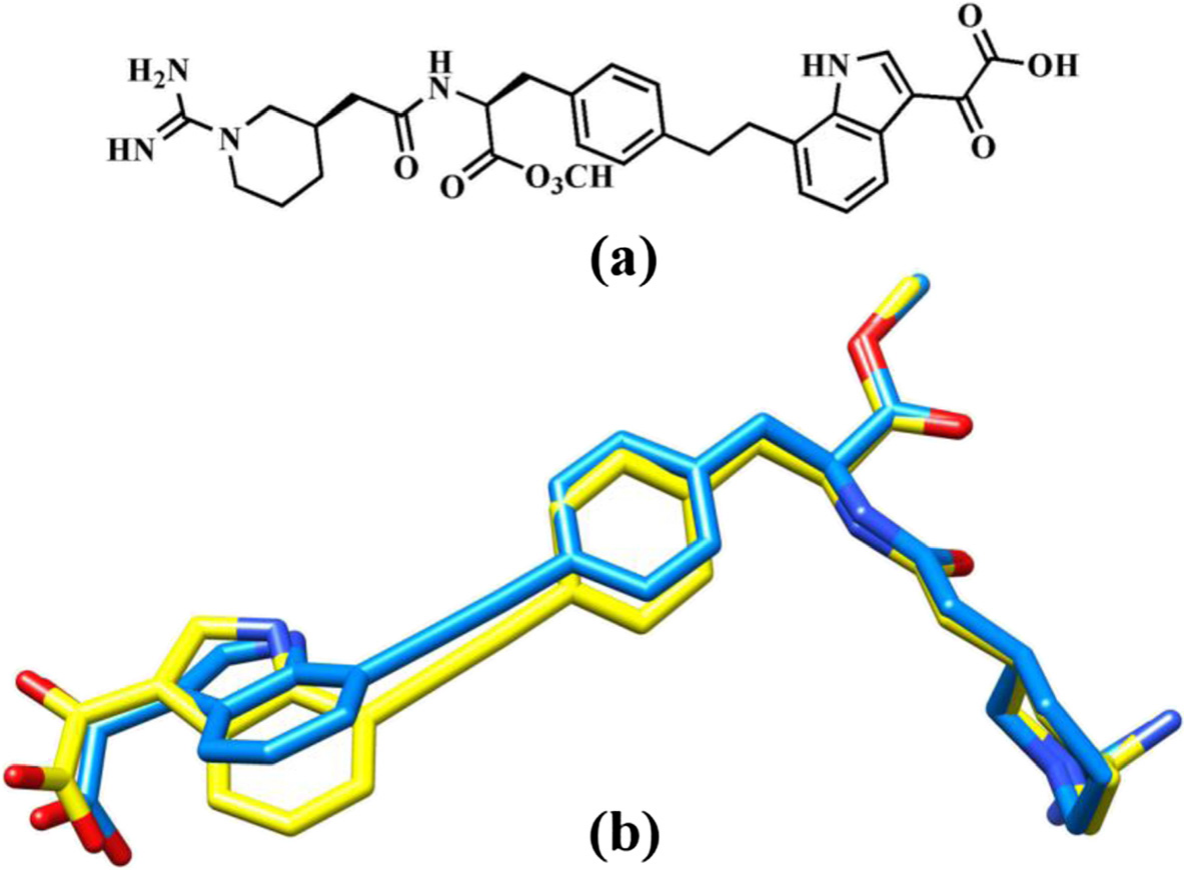
**The chemical structure of compound 4.** Superimposed view of docked conformation of compound 4 (RMSD 0.7 Å), shown in blue **(a)** and the reference ligand shown in yellow **(b)**.

In continuation with our ongoing molecular docking studies on IL-2 [20,21], molecular docking studies were conducted to predict the binding pattern of compounds 1–3 with IL-2. The observed binding modes suggested that all three compounds bind at the ligand binding site of IL-2. Prior to the docking of compounds 1–3, the performance docking program was evaluated through the re-docking experiment. For this purpose, the co-crystallized compound 4 (reference ligand) was extracted from the X-ray structure of protein and re-docked into the ligand binding site using FlexX docking program. The FlexX docking program accurately docked the reference ligand with the RMSD value of 0.7 Å. This shows that FlexX is suitable and can be used for the docking of comp 1–3. The superimposed view of experimental and docked conformation of comp. 4 is presented in Figure 2.

## Discussion

### Transformation

**Scheme 1:**
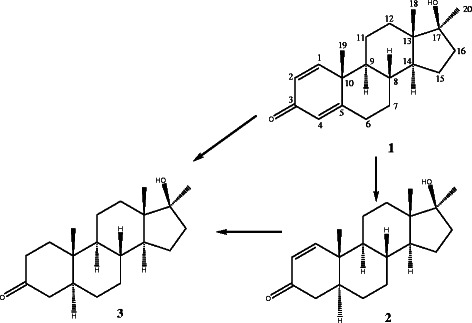
**Biotransformation of dianabol (1) by cell suspension cultures of *Azadirachta indica***, **and resulting metabolites 2 and 3.**

Biotransformation of dianabol (1) by cell suspension culture of *A*. *indica* yielded compounds 2 and 3, (Scheme 1).

Compound 2 was obtained as a colourless solid. The molecular formula C_20_H_30_O_2_ was deduced from the HREI-MS at *m*/*z* 302.0122 (Calcd. 302.0120), indicating six degrees of unsaturation. The IR spectrum showed hydroxyl absorption at 3445, and carbonyl absorption at 1674 cm^-1^. The ^1^H NMR spectrum of compound 2 displayed only one olefinic double bond at *δ*_H_ 7.13, and 5.83 (each d, *J*_1,2_ = 10.2 Hz). The ^13^C NMR spectra also showed disappearance of one olefinic double bond in the compound 2. It showed additional signal of C-4 methylene (*δ*_C_ 41.0), and C-5 methene (*δ*_C_ 44.4). This reduction also confirmed by the HMBC spectrum, in which C-1 (*δ*_H_ 7.13) have *J*_2_ correlations with C-2 (*δ*_C_ 127.5), and C-10 (*δ*_C_ 39.1), and *J*_3_ correaltions with C-3 (*δ*_C_ 200.1) and C-5 (*δ*_C_ 44.4). Similarly, C-5 (*δ*_H_ 1.92-1.94, m) showed *J*_2_ correlations with C-4 (*δ*_C_ 41.0), and C-10 (*δ*_C_ 39.1), and *J*_3_ correlation with C-3 (*δ*_C_ 200.1). The configuration of the newly introduced proton at C-5 was assigned to be *α* on the basis of NOESY correlation between H-5 with CH_3_-20*α*, which showed a *trans* junction between rings A and B. Thus, the structure of compound 2 was identified as 17*β*-hydroxy-17*α*-methyl-5*α*-androst-1-en-3-one [22]. This compound is also known as M1T (methyl-1-testosterone) as well as new designer steroid, and according to a recent article, M1T is featured as a potent androgen by using the yeast AR transactivation assay. It was further demonstrated as potent androgen and anabolic steroid both sc and peroral [23]. M1T is very similar to a well known synthetic anabolic androgenic steroid (AAS); methyltestosterone (MT) [24] a 17α-methylated testosterone [25]. The olefinic double bond(C = C) is at C1/2 in M1T whereas in MT at C4/5 position.

Compound 3 was obtained as a white solid. The HREI-MS of compound 3 showed the *M*^+^ at *m*/*z* 304.2113, corresponding to the formula C_20_H_32_O_2_ (Calcd. 304.2115). The IR spectrum showed a hydroxyl absorption at 3360, and ketonic absorption at 1713 cm^-1^. The reduction of both olefinic double bonds was inferred by the absence of the all the downfield olefinic protons in the ^1^H NMR spectrum of compound 3. The ^13^C NMR spectra of compound 3 also showed three additional upfield methylene and a methine carbon signals i.e. C-1 (*δ*_C_ 38.2), C-2 (39.0), C-4 (44.7) and C-5 (*δ*_C_ 46.8). In the HMBC spectrum, C-19 methyl proton (*δ*_H_ 1.01) showed *J*_2_ correlations with C-10 (*δ*_C_ 35.8), and *J*_3_ correlations with C-1 (*δ*_C_ 38.2), and C-5 (*δ*_C_ 46.8). The configuration of the newly introduced proton at C-5 was assigned to be *α* on the basis of NOESY correlation between H-5 with CH_3_-20*α*, which supported a *trans* junction between rings A and B [26]. Compound 3 is known as mestanolone, a nonionizable pharmaceutical host compounds [27] and the 17α-methylated version of dihydrotestosterone (DHT) [28].

The complete ^1^H [Additional file 1: Table S1] and ^13^C NMR [Additional file 1: Table S2] assignments of compound 2 and 3 are presented in Tables, provided as supporting material. Both of these derivatives are well known androgens and obtained through chemical synthesis. Both M1T (compound 2) and Mestanolone (compound 3) are important intermediate in the synthesis of the anabolic drug oxandrolone [29].

### Biological studies

#### Anti-inflammatory

The activity of compound 2, in contrast to the 1 and 3, could be due to the olefinic bond between C-1/C-2. Compound 3 did not possess a carbon-carbon double bond, and showed lesser activity (Table 1). This suggested that the activity might be due to C-1/C-2 carbon-carbon double bond.

#### Anti-cancer

Compounds 1–3 have similar structures (Scheme 1) except for one and two olefinic double bonds. Compound 2 differs by absence of one and 3 by two olefinic double bonds. Interestingly, compound 2 has a difference of only one olefinic double bonds at C-4 position as compared to its parent compound; probably due to the absence of this double bond compound 3 was not able to inhibit considerable growth of cancer cells at 100 μM that is why this compound was not evaluated further. Compound 1 (substrate), which is different having two olefinic double bond (at C-1 and C-4 positions) exhibited better growth inhibition but not as discriminating as 2, which clearly suggested that absence and presence of olefinic double bond in the given structures is important for having the anticancer activity. Particularly, double bond present at position C-1 is more important than at C-4.

When statistical errors (Table 2) are contemplated for compound 1 and 2 then both compounds would be considered as potent similarly, but because only compound 2 showed total growth inhibition (TGI) that is why this could be designated as most effective compound.

### Docking studies

**Figure 3:**
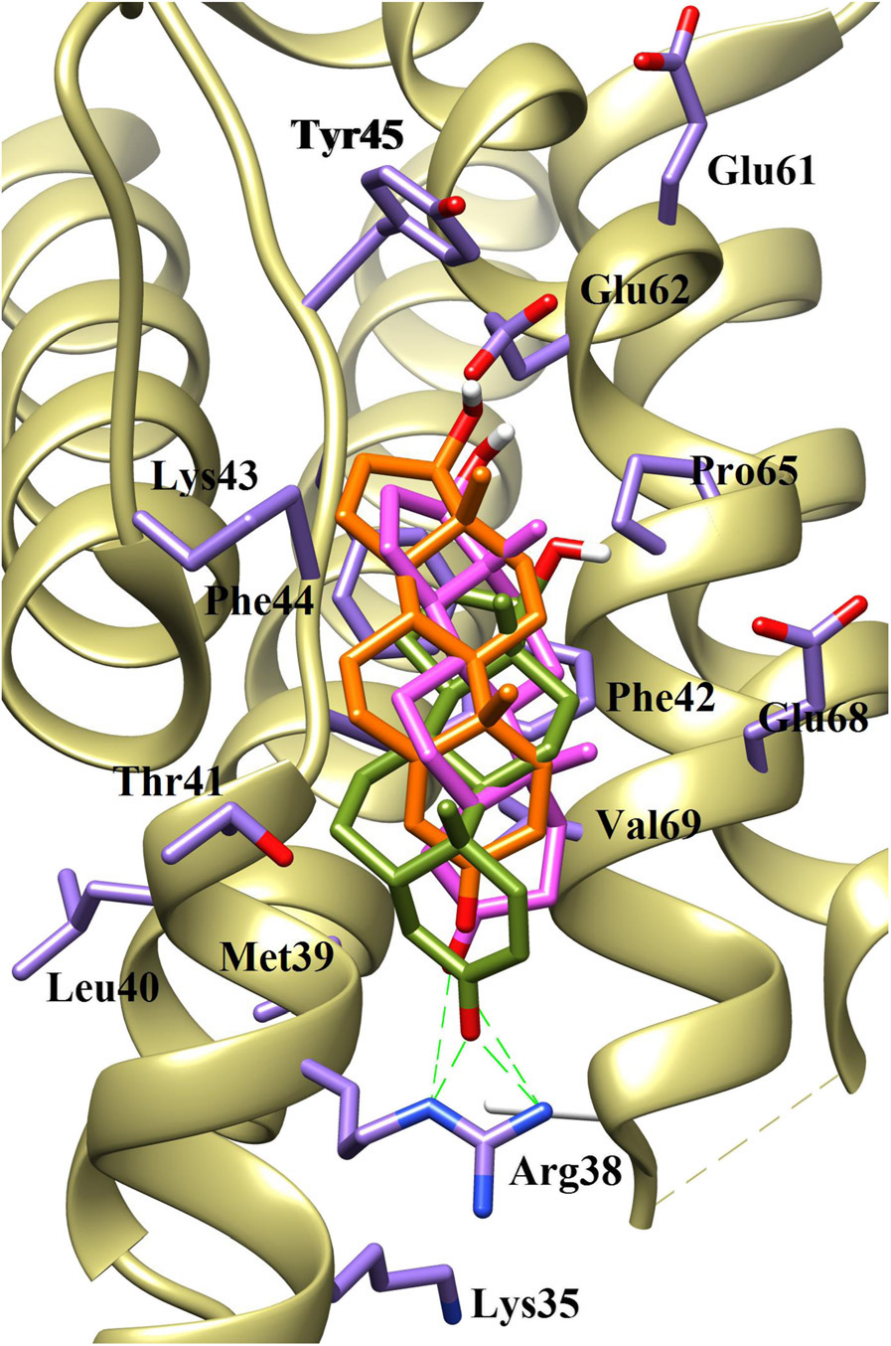
**The docked orientations of Compound 1 (Orange), 2 (Green) and 3 (Pink) in the ligand binding site of IL-2.** The interacting amino acids are shown in Purple stick.

Docking studies showed a good correlation between the predicted binding energies of the compounds obtained by the FlexX program and the experimental binding affinities. Analysis of the docking results revealed that all three compounds bind at the same receptor site on the surface of IL-2 protein. Comparing the binding scores of the compounds 1–3 with the reference ligand (compound 4), it was predicted that compounds 1–3 could inhibit IL-2 protein. All three compounds showed similar binding pattern and interacts on the active site of IL-2 protein at the surface through several active- site amino acid residues (Figure 3).

**Figure 4:**
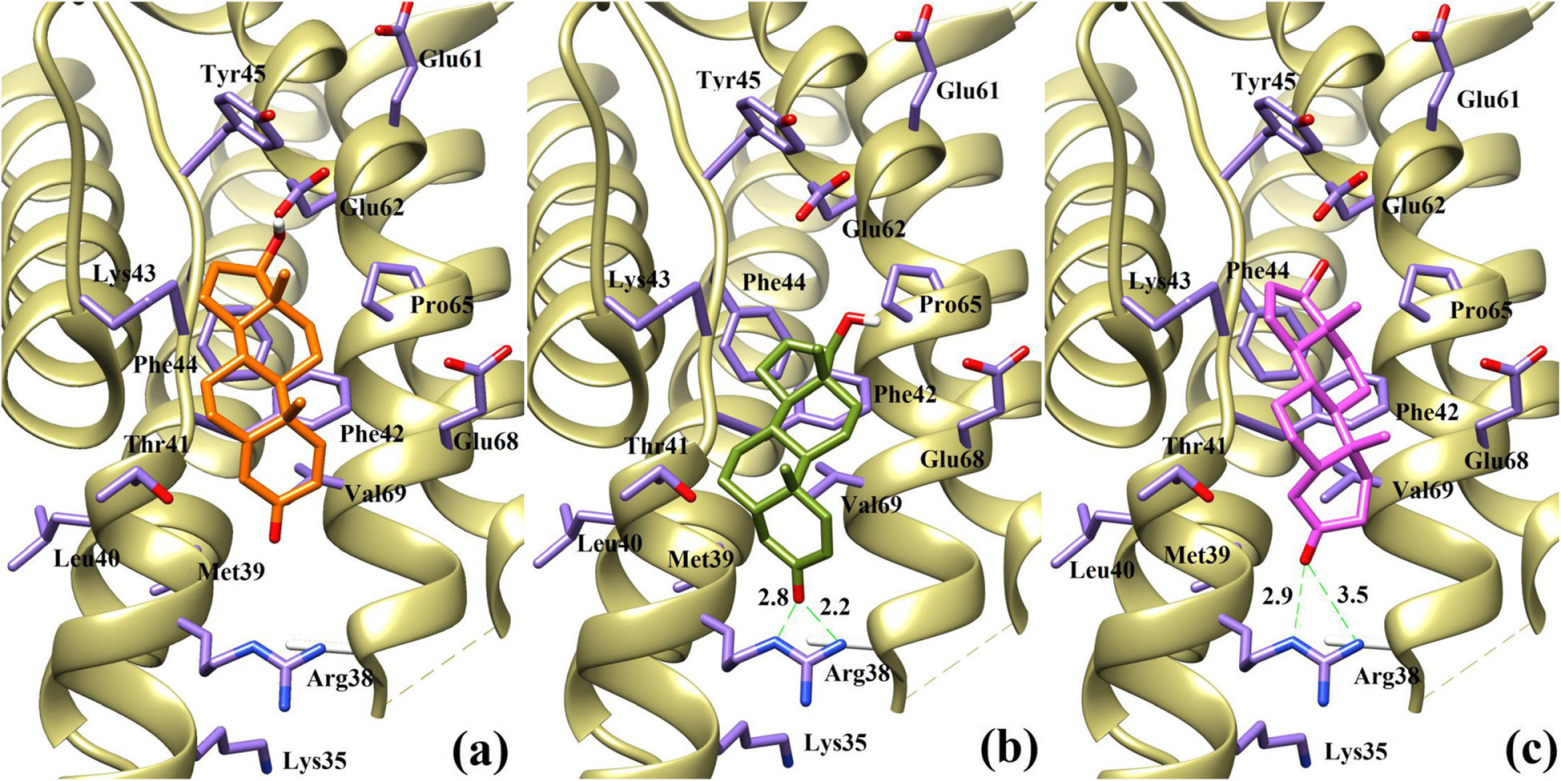
**The binding mode analysis of Compound 1 (a), 2 (b) and 3 (c).** The interaction of compounds 1–3 with Arg38 is shown. Hydrogen bonds are displayed in green dash lines and the distances are shown in red colour.

Compound 1 was least active due to the presence of two olefinic double bonds in conjugation with the carbonyl group of ring A at C-3. The lone pair of the carbonyl group is least likely to be available to interact with surrounding amino acid residues due to conjugation. The observed binding mode of compound 1 is shown in Figure 4a, which shows that compound 1 is unable to form any hydrogen bonding interaction with any surrounding amino acid. This could be a reason of inactivity of this compound. While in compound 2 the carbonyl group of ring A forms bidentate interaction with the side chain guanidinium moiety of Arg38. The side chain Nϵ and NH2ŋ^2^ of Arg38 forms hydrogen bonding with the carbonyl moiety at distance of 2.8 Å and 2.2 Å, respectively (Figure 4b). The binding mode analysis of compound 3 shows that, compound 3 also makes weak bidentate interaction with Arg38 in comparison to compound 2. The observed hydrogen bond distance between Nϵ and NH2ŋ^2^ of Arg38 and compound 3 was 2.9 Å and 3.5 Å, respectively (Figure 4c). Due to this weak interaction, compound 3 is less active than compound 2. The binding modes of all three compounds (1–3) are shown in Figure 4. All three compounds are additionally stabilized by a number of hydrophobic interactions offered by Met39, Thr41, Phe42, Phe44, Pro65 and Val69 at the A’ B loop of the IL-2. Our docking results revealed the importance of Arg38 in the vicinity of carbonyl group of ring A, which plays a vital role in protein-ligand complex formation and stabilization.

**Figure 5:**
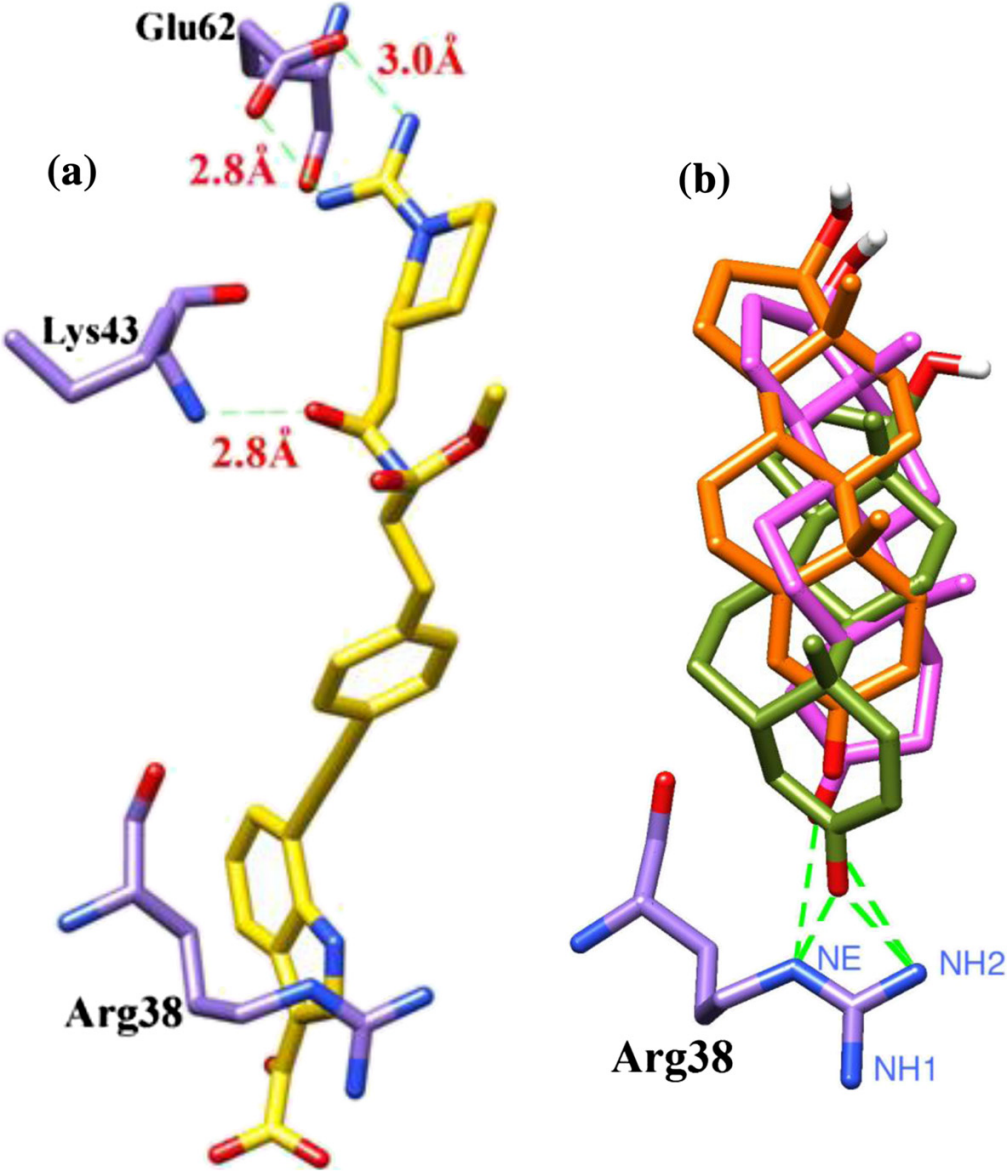
**The comparison of hydrogen bonding pattern of compounds 1–3 with reference compound 4.** Hydrogen bonding of reference compound with Arg38 **(a)** and Hydrogen bonding of compound 1-3 with Arg38 **(b)**.

The hydrogen bonding pattern of compounds 1–3 were compared with that of reference ligand 4. The guanidinium moiety of Compound 4 mediates bidentate interaction with the side chain of Glu62 while the peptide carbonyl interacts with the backbone amino group of Lys43. Compounds 1–3 pose only one hydrogen bond donor feature i.e. hydroxyl group at C17 of ring D, which is surface exposed and do not contribute in protein-ligand interaction, while carbonyl group interacts with Arg38 instead of Lys43. The hydrogen bonding patterns of compounds 1–3 are compared with the hydrogen bonding pattern of compound 4 in Figure 5.

## Methods

### General methods

The ^1^H-NMR spectra were recorded in CDCl_3_ on Bruker AM-300 and AM-400 NMR spectrometers with TMS as an internal standard using UNIX operating system at 300 and 400 MHz, respectively. The ^13^C-NMR spectra were recorded in CDCl_3_ at 100 MHz on Bruker AM-400 NMR spectrometer. HREI-MS were recorded on Jeol JMS 600 and HX 110 mass spectrometers with the data system DA 5000. The IR spectra were recorded on a Jasco A-302 spectrophotometer. The UV spectra were recorded on a Hitachi U-3200 spectrophotometer. The optical rotations were measured on JASCO DIP-360 digital polarimeter. The melting point was determined on a Buchi 510 apparatus. Column chromatography (CC) was carried on silica gel column (70–230 Mesh). Purity of the samples was checked by TLC on pre-coated silica gel GF-254 preparative plates (20 × 20 cm, 0.25 mm thick, Merck) and were detected under the UV lights (254 and 366 nm), while ceric sulphate was used as spraying reagent. Dianabol (1) was purchased from Fluka Riedel-deHaën® and the purity was 99.0% (HPLC).

### Callus culture

The plant was identified by the department of Botany, University of Karachi. The callus cultures of the plant were derived from young leaves cultivated in 300 mL jars, each having 25 mL of Murashige and Skoog medium [30], supplemented with sucrose (30 g/L), 3-indole butyric acid (IBA) (19.7 μM), and 6-benzyl aminopurine (BA) (4.44 μM), and solidified by agar (6 g/L) and incubated at 25 ±1°C under complete darkness.

### Biotransformation protocol

Cell suspension cultures were derived from static cultured calli in Erlenmeyer flasks (1000 mL), each containing 400 mL of the Murashige and Skoog medium, supplemented with ingredients as mentioned above except BA and agar. After 15 days of pre-culturing on a gyratory platform shaker at 100 rpm and with a 16 h photoperiod at 25 ±1°C, a solution of substrate (100 mg in 1 mL of acetone) was added to each flask through a 0.2 μM membrane filter and the flasks were placed on a shaker for 20 days. The time course study was carried out by taking aliquots from culture on daily basis and the content of transformation was analyzed by TLC. A negative control containing only plant cell suspension cultures and a positive control containing compound 1 in the media were also prepared in order to check the presence of plant metabolites in the cell culture, and the chemical changes as a result of chemical reaction (if any) due to media components, respectively.

### Immunomodulatory studies

#### Reagents, chemicals, and equipments

Luminol (3-aminophthalhydrazide), Hanks Balance Salts Solution (HBSS) and Lymphocytes Separation Medium (LSM) were purchased from Research Organics, Sigma, Germany, and MP Biomedicals, Inc., Germany, respectively. Zymosan-A (*Saccharomyces cerevisiae* origin), Dimethylsulfoxide (DMSO), ethanol and ammonium chloride of analytical grades were purchased from Merck Chemicals, Darmstadt, Germany. The Luminometer used was Luminoskan RS, Finland.

#### Isolation of human polymorphonuclear cells (PMNs)

Heparinized blood was obtained by vein puncture aseptically from healthy volunteers (25 – 38 years age). The buffy coat containing PMNs was collected by dextran sedimentation and the cells were isolated after the LSM density gradient centrifugation from the tube base. Cells were washed twice and suspended in Hank’s Balance Salt Solution (Ca^2+^ and Mg^2+^ free) (HBSS^--^), pH 7.4. Neutrophils were purified from RBCs (red blood cells) contamination using hypotonic solution. Cells were adjusted to their required concentration using Hank’s Balance Salt Solution containing Ca^2+^ and Mg^2+^ (HBSS^++^).

### Oxidative burst study

Luminol -enhanced chemiluminescence assay was performed as described by Helfand *et al*. [31] and Haklar *et al*. [32] with some modifications. Briefly, 25 μL diluted whole blood (1:200 dilutions in sterile HBSS^++^) or 25 μL of PMNs (1 × 10^6^) cells were incubated with 25 μL of serially diluted compounds with concentration ranges between 3.2–50 mg/mL. Tests were performed in white 96-well plates, which were incubated at 37°C for 30 minutes in the thermostatic chamber of the luminometer. Opsonised zymosan-A, 25 μL followed by 25 μL luminol (7 × 10^-5^ M) along with HBSS^++^ was added to each well to obtain a 100 μL volume/well. Wells received HBSS^++^ and cells but no compounds were used as a negative control. Chemiluminescence peaks were recorded with the Luminometer. Results were monitored as chemiluminescence relative light units (RLU) with peak and total integral values.

### T- Cell proliferation assay

The T-cell proliferation assay was performed as described by Nielsen *et al*. [33]. In this assay, isolated lymphocytes were stimulated by adding the phytohemagglutinin (PHA) in culture. The rate of proliferation and survival was measured by radio-labelled thymidine incorporation method. The lymphocytes were isolated as described by Boyum [34]. A 50 μL of cell suspension (10^6^/mL) was added to each well of a 96-well round bottom tissue culture plate. 50 μL of PHA was added in each well from the working solution (20 μg/mL) to have final concentration 5 μg/mL. The final volume was adjusted to 200 μL in each well by adding complete RPMI media. The plates were incubated at 37˚C for 72 hours (90% humidity, 5% CO_2_ /air). Methyl-^3^H thymidine (Amersham Pharmacia Biotech) 0.5 μCi was added in each well and incubated for further 18 hours. Cells were harvested on a filter mat (Type G-7) using cell harvester (INOTECH IH-280, Switzerland) and the radioactivity (CPM) was measured using a β-scintillation counter (LS 6500, Beckman Coulter, USA). The control drug prednisolone was purchased from Sigma Aldrich^™^.

### IL-2 Cytokine production

IL-2 cytokine was produced from peripheral blood mononuclear cells (PBMC). IL-2 cytokine production by PHA activated cells in the presence or absence of test compounds was studied by ELISA using the human cytokine kit (Diaclone, Besancon Cedex France). Briefly, freshly prepared mononuclear cells (10^5^/well) were cultured in 96-well microtiter flat bottom plate in the presence or absence of 5 μg/mL PHA. Four different concentrations (10.33-331.13 μM/mL) of compounds, along with PHA, were used in this assay. The culture plate was incubated at 37°C for 18 h. Then the supernatant was collected and analyzed for IL-2 cytokine production following kit manufacturer instructions. Results of all above test are presented in Table 1.

### Statistical analysis

Students T-test was performed to compare the significance mean differences between the control and tested extracts for various chemiluminescence results. The differences were considered to be significant at levels of P ≤ 0.05.

### Anticancer activity

The sulforhodamine B (SRB) protein staining assay was employed for measurement of *in vitro* growth inhibition and cytotoxicity [35]. The appropriate cell density (cells/well) of NCI-H460 cells (1 × 10^4^) was added in 96-well plates. The cell density used was dependent on the doubling time of the cell line leading to the formation of monolayer. After 24 h incubation, different doses of compounds 1–3 were added and incubated for further 48 h. This was followed by fixation of cells with ice-cold trichloroacetic acid (50 μl, 50%) at room temperature for 30 min. The plates were carefully washed five times with distilled water, and left for overnight drying in air. Sulforhodamine B dye (100 μl, 0.4% in 1% acetic acid) was introduced in each well and after 30 min residual dye was removed using acetic acid (1%) and air-dried overnight. The bound SRB dye was solubilised in Tris-base solution (100 μl, 10 mM) with gentle shaking on a plate-shaker for 5 min prior to optical density (OD) measurements at 545 nm in a plate reader. From the optical densities values, the percentage growths inhibition was calculated using the following formulae:

IfT≥Tz,

$$\begin{array}{l} \mathrm{the}\;\mathrm{Percentage}\;\mathrm{growth}\;\mathrm{of}\;\mathrm{cells}\\ \;=\;100\left[\left(\mathrm{T-Tz}\right)/\left(\mathrm{C-Tz}\right)\right] \end{array}$$

IfT<Tz,

$$\begin{array}{l} \mathrm{the}\;\mathrm{Percentage}\;\mathrm{growth}\;\mathrm{of}\;\mathrm{cells}\\ \;=\;100\left[\left(\mathrm{T-Tz}\right)/\mathrm{Tz}\right] \end{array}$$

where T and Tz indicates the absorbance values at the time cells received the tested compound (Tz) and after a period of treatment (T), C indicates the absorbance value measured in untreated cells (control) after an incubation period equal to the treatment period.

Percentgrowthinhibition=100%-percentagegrowthofcells

From the percentage growth, a dose response curve was generated between the sample concentration and percent growth of the cells to determine GI_50_ and TGI LC_50_ parameters.

Where GI_50_ parameter indicates the sample concentration for growth inhibition (50%) of total cells. TGI indicates the sample concentration for complete growth inhibition (100%) of the cells.

### Molecular docking protocol

The three dimensional structure of Human IL-2 in complex with compound 4 [2-[2-(1-Carbamimidoyl-Piperdin-3-yl)-acetylamino]-3-{4-[2-(3-Oxalyl-1H-Indol-7-yl) ethyl] phe -nyl}-propionic acid methyl ester] was downloaded from protein data bank [PDB ID: 1 M49; resolution 2.0 Å]. Docking protocol was evaluated through re-docking process in which the co-crystallized compound was extracted and re-docked into the binding cavity, and the quality of docking protocol was evaluated through root mean square deviation (RMSD ≤2 Å is considered as best). In this study, Compound 4 was used as a reference for docking studies. Compound 4 was synthesized by Tilley *et al*. [36], and its chemical characterization is reported. The compound was co-crystallized with IL-2 by Arkin *et al*., [37]. The re-docking of compound 4, suggested that the docking protocol is suitable for the docking analysis of comp. 1–3.

The three-dimensional structures of compounds 1–3 were generated by molecular modeling software SYBYL 6.9 [38]. Energy minimization was carried out using the Tripos force field with a distance gradient algorithm with a convergence criterion of 0.05 kCal/ (molÅ) and maximum 10,000 iterations, respectively, with Gasteiger–ǘckel charges (TRIPOS Inc) [39].

Docking of ligand 1–3 into the active site of IL-2 receptor was performed using FlexX docking software implemented in SYBYL6.9. FlexX software is a fast and flexible algorithm for docking small ligand into binding sites of the enzymes, using an incremental construction algorithm that actually builds the ligand in the binding site [40]. The software incorporates protein–ligand interactions, placement of the ligand core, and rebuilding of the complete ligand. A receptor description file (RDF) was created from the PDB coordinates. The active site for docking was defined as all atoms within 6.5 Å radius of the co-crystallized ligand 4. The proposed interaction mode of the ligand in the active-site of IL-2 was determined as the highest scored conformation (best fit ligand) among 30-generated conformations and binding modes generated according to FlexX scoring function, which is the structure with the most favourable free energy of binding. Docking results were analyzed by VMD (visual molecular dynamics) [41]. Docking energies and H-Bond distances with the Arg38 are presented in Table 3.

**Table 3:** Docking Energies and H-Bond Distances with the surrounding residues

**Compounds**	**Binding**	**H-bond**		
	**scores**	**distance (Å)**		
		**Arg38**	**Lys43**	**Glu62**
1	−6.7	-----	------	------
2	−10.8	2.8 (NH2ϵ) and 2.2 (NH2ŋ^2^)	------	------
3	−9.8	2.9 Å (NH2ϵ) and 3.5 Å (NH2ŋ^2^)	------	------
4	−26.0	------	2.9 Å (NH2)	2.8 Å (O_1_) and 3.0 Å (O_2_)
Prednisolone	−28.2	-----	2.44	3.34

## Conclusion

Biotransformational studies resulted in the production of two metabolites, 2 and 3 (first time using this way) when cell suspension culture of *A*. *indica* was incubated with compound 1. Metabolite 2 exhibited potent and differential *in*-*vitro* immunomodulatory activity particularly against T-cell proliferation; this was further resulted into significantly interfere with IL-2 protein. Comparably, metabolite 3 and substrate (1) did not show considerable results. Compound 2 also demonstrated a moderate anti-cancerous activity slightly higher than the parent compound but no activity was observed for metabolite 3. All the compounds (1–3) were found similar according to their binding pattern to IL-2 protein in molecular docking studies but the binding score was lowest for metabolite 2.

## Competing interests

Saifullah Khan and Muhammad Ahmed Mesaik acknowledge the financial support of EMRO-COMSTECH and HEC for successful executing the study, respectively.

## Authors’ contributions

Saifullah performed all the experiments, prepared and corrected manuscript. SK: supervised all the tissue culture related experiments. A: elucidated the structures of the compounds. SAH: carried out whole docking studies. MK: participated in anti-cancer activity performance. AJ: executed T-cell and IL-2 assays. MA: studied whole blood and PMN activities. MAM: supervised anti-inflammatory methods and interpretation. ZUH: supervised and interpreted docking studies. AD: corrected and supervised anti-cancer assay. MIC: helped and supervised elucidating spectroscopic data. All authors read and approved the final manuscript.

## Supplementary Material

Additional file 1 Table S1 ^1^H NMR (300 MHz, CDCl_3_)^a^) chemical shifts of compound 1 and its metabolites 2 and 3. δ in ppm and *J* in Hz. **Table S2. **^13^C NMR (100 MHz, CDCl_3_)^a^)^b^) chemical shifts of compound 1 and its metabolites 2 and 3. Click here for file
