# Supplementary material for: In-vitro immunomodulatory and anti-cancerous activities of biotransformed products of Dianabol through Azadirachta indica and its molecular docking studies
Source: Chem Cent J. 2013 Oct 7;7:163. doi: 10.1186/1752-153X-7-163 (PMC3874778; doi:10.1186/1752-153X-7-163)
Supplement: Additional file 1 Table S1 — 1H NMR (300 MHz, CDCl3)a) chemical shifts of compound 1 and its metabolites 2 and 3. δ in ppm and J in Hz. Table S2. 13C NMR (100 MHz, CDCl3)a)b) chemical shifts of compound 1 and its metabolites 2 and 3. [file 1752-153X-7-163-S1.doc]

**Table 1:** ^1^H NMR (300 MHz, CDCl_3_)^a^) chemical shifts of compound **1** and its

metabolites **2** and **3**. δ in ppm and *J* in Hz.

| **C. NO.** | **1** | **2** | **3** |
| --- | --- | --- | --- |
| 1 | 7.03 (d, *J*_1,2_ = 10.2) | 7.13 (d, *J*_1,2_ = 10.2) | 2.05-2.08 (m)^b^  2.40-2.42 (m) |
| 2 | 6.19 (dd, *J*_2,1_ = 10.2, *J*_2,4_ = 1.9) | 5.83 (d, *J*_2,1_ = 10.2) | 1.68-1.70 (m)  1.80-1.82 (m) |
| 3 | - | - | - |
| 4 | 6.04 (br. s) | 2.20-2.23 (m)  2.37-2.40 (m) | 2.06-2.08 (m)^b^  2.24-2.26 (m) |
| 5 | - | 1.92-1.94 (m) | 1.93-1.95 (m) |
| 6 | 2.32-2.34 (m)  2.47-2.50 (m) | 2.28-2.30 (m)  2.43-2.45 (m) | 2.30-2.32 (m)  2.31-2.34 (m) |
| 7 | 1.28-1.30 (m)  1.62-1.64 (m)^b^ | 1.32-1.35 (m)  1.56-1.58 (m) | 1.30-1.32 (m)  1.49-1.52 (m) |
| 8β | 1.67-1.70 (m)^c^ | 1.50-1.52 (m) | 1.47-1.50 (m) |
| 9α | 1.00-1.02 (m) | 1.15-1.18 (m) | 0.92-0.95 (m) |
| 10 | - | - | - |
| 11 | 1.62-1.64 (m)^b^  1.64-1.67 (m) | 1.63-1.65 (m)^b^  1.78-1.80 (m) | 1.51-1.53 (m)  1.62-1.65 (m) |
| 12 | 1.20-1.22 (m)  1.53-1.56 (m)  1.62 | 1.21-1.23 (m)  1.62-1.64 (m)^b^  1.62 | 1.18-1.20 (m)^c^  1.42-1.44 (m)  1.62 |
| 13 | - | - | - |
| 14α | 1.15-1.18 (m) | 1.12-1.14 (m) | 1.18-1.20 (m)^c^ |
| 15 | 1.30-1.32 (m)  1.32-1.35 (m) | 1.41-1.44 (m)^c^  1.43-1.45 (m)^c^ | 1.36-1.38 (m)  1.38-1.40 (m) |
| 16 | 1.68-1.70 (m)^c^  1.81-1.83 (m)  1.89 | 1.71-1.73 (m)  1.82-1.85 (m)  1.89 | 1.64-1.66 (m)  1.82-1.84 (m)  1.89 |
| 17 | - | - | - |
| 18 | 0.91 (s) | 0.87 (s) | 0.85 (s) |
| 19 | 1.22 (s) | 1.01 (s) | 1.01 (s) |
| 20 | 1.16 (s) | 1.20 (s) | 1.19 (s) |

*^a)^ assignments based on COSY and HMQC.*

*^b,c)^ signals may be interchanged.*

**Table 2:** ^13^C NMR (100 MHz, CDCl_3_)^a^)^b^) chemical shifts of compound **1** and its metabolites **2** and **3**.

| **C. NO.** | **1** | **2** | **3** |
| --- | --- | --- | --- |
| 1 | 155.7 *(d)* | 158.3 *(d)* | 38.2 *(t)* |
| 2 | 127.5 *(d)* | 127.5 *(d)* | 39.0 *(t)* |
| 3 | 186.2 *(s)* | 200.1 *(s)* | 211.9 *(s)* |
| 4 | 123.8 *(d)* | 41.0 *(t)* | 44.7 *(t)* |
| 5 | 169.0 *(s)* | 44.4 *(d)* | 46.8 *(d)* |
| 6 | 32.8 *(t)* | 27.6 *(t)* | 28.9 *(t)* |
| 7 | 33.3 *(t)* | 31.6 *(t)* | 31.6 *(t)* |
| 8 | 36.4 *(d)* | 36.6 *(d)* | 36.2 *(d)* |
| 9 | 52.5 *(d)* | 50.7 *(d)* | 53.9 *(d)* |
| 10 | 43.6 *(s)* | 39.1 *(s)* | 35.8 *(s)* |
| 11 | 22.5 *(t)* | 20.9 *(t)* | 21.1 *(t)* |
| 12 | 31.3 *(t)* | 31.0 *(t)* | 31.5 *(t)* |
| 13 | 45.6 *(s)* | 45.7 *(s)* | 45.6 *(s)* |
| 14 | 49.8 *(d)* | 50.1 *(d)* | 50.6 *(d)* |
| 15 | 23.3 *(t)* | 23.2 *(t)* | 23.3 *(t)* |
| 16 | 38.7 *(t)* | 39.0 *(t)* | 38.6 *(t)* |
| 17 | 81.3 *(s)* | 81.5 *(s)* | 81.6 *(s)* |
| 18 | 13.9 *(q)* | 14.1 *(q)* | 14.0 *(q)* |
| 19 | 18.7 *(q)* | 13.1 *(q)* | 11.5 *(q)* |
| 20 | 25.8 *(q)* | 25.9 *(q)* | 25.8 *(q)* |

*^a^) multiplicities were determined by DEPT experiments.*

*^b^) assignment based on HMQC and HMBC.*
